# Occurrence of Co-Contamination and Interaction of Multi-Mycotoxins in Dairy Cow Feed in China

**DOI:** 10.3390/toxins17030137

**Published:** 2025-03-14

**Authors:** Zuoyin Zhu, Haisheng Cheng, Jie Wang, Junmei Ma, Jianhua Wang, Hongyang Wang, Xinli Zhou, Junhua Yang

**Affiliations:** 1 School of Health Science and Engineering, University of Shanghai for Science and Technology, Shanghai 200093, China; zhuzuoyin123@163.com (Z.Z.);; 2 Institute for Agro-Food Standards and Testing Technology, Shanghai Academy of Agricultural Sciences, Shanghai 201403, China; jianhuawang163@163.com; 3 Xining Agricultural Product Quality and Safety Testing Center, Xining 810003, China; 4 Institute of Animal Science and Veterinary Medicine, Shanghai Academy of Agricultural Sciences, Shanghai 201106, China

**Keywords:** feed, mycotoxin, co-contamination, correlation, interaction

## Abstract

Co-contamination of multiple mycotoxins in feed has become one of the most important issues in the world. In this study, the characteristics and interactions of co-contamination among 15 mycotoxins were explored in dairy cow feed, including total mixed ration (TMR), silage, maize, and hay feed. The results showed that four dairy cow feeds were constantly contaminated with mycotoxins, including zearalenone (ZEN), fumonisins (FBs), deoxynivalenol (DON), ochratoxin A (OTA), T-2 toxin (T-2), and aflatoxins (AFs). The contamination level of each mycotoxin was low, but the probability of co-contamination by three or more mycotoxins in one sample was very high. Between DON and aflatoxin B2 (AFB2), between aflatoxin M1 (AFM1) and OTA, between FB2 and aflatoxin B1 (AFB1), between 15-acetyl-deoxynivalenol (15-ADON) and ZEN, and between fumonisin B1 (FB1) and fumonisin B3 (FB3), and between aflatoxin M2 (AFM2) and aflatoxin G2 (AFG2), there were significant and strong correlations. Among the four typical feed samples, the combinations DON + ZEN, DON + FB1, FB1 + ZEN, OTA + ZEN, DON + 3-acetyl-deoxynivalenol (3-ADON), 3-ADON + ZEN, T-2 + ZEN, fumonisin B2 (FB2) + ZEN, and DON + FB3 had higher interaction rates than the other combinations (≥43.75%). Our study not only reveals that co-contamination with multiple mycotoxins is relatively common in dairy cow feed but also highlights the significant correlations between various mycotoxins and assesses the likelihood of their interactions. These findings are crucial for ensuring feed safety and safeguarding animal health.

## 1. Introduction

Alongside China’s rapid economic development and the continuous improvement in living standards for both urban and rural residents, dairy products have emerged as an essential component of the Chinese diet [1]. However, the feed is an important resource to guarantee the safety and quality of dairy products and the development of animal husbandry [2]. Dairy cow feed can be divided into silage made from grass and grain crops, high-energy or high-protein feed made from grains and other self-produced crops, and compound feed mixed with a single feed supplemented with other nutrients [3,4]. Forage grass, wheat, maize, and other crops are the most important part of the animals’ daily diet and the most important component of compound animal feed. Moreover, half of the maize production and one-fifth of wheat production in the world are used to produce animal feed [5,6].

Mycotoxins are toxic metabolites generated by fungi [7], which are often combined in crop feed and are also the main sources of ruminants’ mycotoxin intake. In the rumen, the microbiota of ruminants can effectively degrade or inactivate mycotoxins ingested from the diet to mitigate the toxicity of mycotoxins [8]. However, the ability of rumen microbiota to detoxify substances is limited, and ruminants ingesting mycotoxin-contaminated feeds for long periods of time still experience unpredictable health risks. According to the contaminated extent of mycotoxins and toxic effects on health, there are about 400 mycotoxins that have been identified and reported, and among which the aflatoxins (AFs), deoxynivalenol (DON), fumonisins (FBs), ochratoxin A (OTA), zearalenone (ZEN), and T-2 toxin (T-2) are considered the major toxins that endanger animal health and create economic problems [9,10,11,12,13]. A growing body of research has documented that mycotoxin exposure in humans and animals could induce poisoning, teratogenicity, and other health risks. For instance, AFB1 is a serious carcinogenic toxin classified as a Class 1 carcinogen by the World Health Organization’s International Agency for Research on Cancer (IARC), and OTA, fumonisin B1 (FB1), and fumonisin B1 (FB2) are classified as Group 2B carcinogen (possibly carcinogenic to humans) [14,15]. Several countries and organizations around the world have issued limits for the mycotoxin level in feed. However, the maximum level of these mycotoxins only considers single-toxin exposure.

In general, there are several kinds of mycotoxins that co-occur in feed, and which are more common for animals treated with multiple mycotoxins at low doses than single mycotoxin at high doses. The common exposure of toxins can be attributed to one kind of fungi producing several mycotoxins and feed that is polluted simultaneously by several kinds of fungi [16]. Therefore, mixed feed for dairy cows is particularly susceptible to multiple mycotoxin contamination, because it is a mixture of multiple raw materials. Literature surveys showed that the combination of various mycotoxins would cause adverse effects on cells and animals [17,18]. The toxicity of mycotoxins coexisting is not predicted according to the toxicity of a single toxin, because the interaction of multi-mycotoxins may present additive, synergistic, and antagonistic effects. Therefore, the co-contamination of mycotoxins in feed deserves more attention and research.

Dairy cows are one of the species that are sensitive to mycotoxins in animals, and the co-contamination of mycotoxins in dairy cow feed causes a potential public health threat in China. The current study employed validated ultra-performance liquid chromatography-tandem mass spectrometry (UPLC-MS/MS) to detect 15 mycotoxins’ co-contamination in four typical feeds including total mixed ration (TMR), silage, maize, and hay feed, and analyzed the condition and characteristics of the multi-mycotoxins (AFB1, aflatoxin B2 (AFB2), aflatoxin G1 (AFG1), aflatoxin G2 (AFG2), aflatoxin M1 (AFM1), aflatoxin M2 (AFM2), DON, 3-acetyl-deoxynivalenol (3-ADON), 15-acetyl-deoxynivalenol (15-ADON), FB1, FB2, fumonisin B3 (FB3), T-2, OTA, and ZEN) using correlation. Additionally, the interaction rates and co-contamination of mycotoxins in dairy cattle feed were analyzed using a Java-based program and a co-contamination index. Finally, the aim was to generate essential data for mycotoxin safety assessments in dairy cattle feed and to establish a scientific basis for developing more effective control strategies.

## 2. Results

### 2.1. Performance of the Applied Analytical Method

Method validation was conducted in accordance with the guidelines outlined in the European Commission Decision (2023/2782/EC). The linear range, R^2^ values, limit of detection (LOD), and limit of quantification (LOQ) for the method are presented in Table 1. The results indicated that the standard curves for all 15 mycotoxins exhibited strong linearity (R^2^ ≥ 0.9921), with detection limits ranging from 0.04 to 5 μg/kg. Validation results are provided in Table 2, showing that the average recoveries ranged from 70% to 120% across three spiked levels in the four matrices.

### 2.2. Occurrence of Multiple Mycotoxins

**Figure 1:**
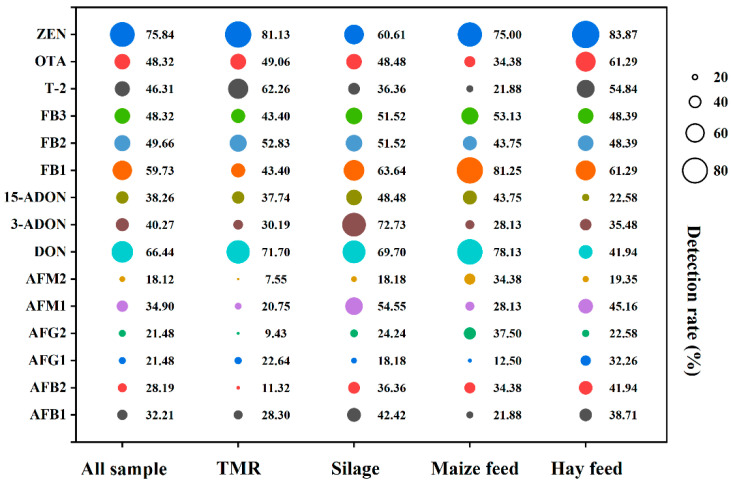
The overall detection rates of 15 mycotoxins in all samples, TMR, silage, maize, and hay feed. The size of the circle indicates the detection rate of mycotoxins.

As shown in Figure 1, 15 mycotoxins were detected in all 149 samples to different extents. It is worth noting that mycotoxins were detected in all feed samples, with varying levels of contamination. For all samples (*n* = 149), ZEN had the highest detection rate (75.84%), followed by DON (66.44%), FB1 (59.73%), FB2 (49.66%), FB3 and OTA (48.32%), T-2 (46.31%), 3-ADON (40.27%), 15-ADON (38.26%), AFM1 (34.90%), AFB1 (32.21%), AFB2 (28.19%), AFG1 and AFG2 (21.48%), and AFM2 (18.12%). In the TMR samples (*n* = 53), ZEN had the highest detection rate of 81.13%, followed by DON (71.70%), T-2 (62.26%), and FB2 (52.83%); the detection rates of other mycotoxins ranged from 7.55% to 49.06%. In the silage samples (*n* = 33), 3-ADON (72.73%) was the most detected toxin, followed by DON (69.70%), ZEN at 60.61%, and AFM1 (54.55%); the other mycotoxins were detected with probabilities ranging from 18.18% to 51.52%. In the maize feed samples (*n* = 32), FB1 (81.25%) had the highest detection rate amongst the mycotoxins. DON was detected in 78.13%, followed by ZEN in 75.00% and FB3 in 53.13% of samples. In the hay feed samples (*n* = 31), ZEN (83.87%) was detected at the highest rate, followed by FB1 and OTA (61.29%), T-2 at 54.84%; the other mycotoxins were detected at probabilities ranging from 19.35% to 48.39%.

### 2.3. Contamination Levels of Mycotoxins

In the most samples, three to seven different mycotoxins were detected, and the results are shown in Table 3. The average contamination in TMR was as follows: AFs, T-2, 3-ADON, 15-ADON, FB2, FB3, and OTA, with average values from 0.13 to 14.05 μg/kg. The average contamination of DON was 32.63 μg/kg. Furthermore, the average contamination amount of FB1 was 142.81 μg/kg, and that of ZEN was 70.61 μg/kg, and the range of contamination was 0.63–696.18 μg/kg. In silage samples, the average value of FB1 was 62.40 μg/kg, and that of 3-ADON was 77.65 μg/kg, while the other mycotoxins had contamination levels below 14.86 μg/kg. In feed samples of maize, the average level of FB1 was 58.28 μg/kg and contamination ranged from 2.37 to 430.79 μg/kg. On average, the level of DON was 36.75 μg/kg, and DON, 3-ADON and 15-ADON contamination was in the range from 0.14 to 476.24 μg/kg. However, the other 13 mycotoxins had contamination levels below 12.20 μg/kg. In hay feed samples, the average contamination of FB1 was 70.24 μg/kg, with contamination ranging from 0.24 to 584.06 μg/kg, and that of FB2 was 21.81 μg/kg, and that of the remaining 13 mycotoxins was 0.27 to 13.94 μg/kg, which was relatively low. According to the Chinese feed hygiene standard (No. GB 13078-2017), only two silage samples exceeded the Chinese Maximum Residue Limits (MRLs) for AFB1, with a 6.06% exceedance rate and concentrations of 42.11 and 53.69 μg/kg, respectively. The average contamination levels of all other mycotoxins were well below the national safety limit, and the risk level was also low (Table S1).

### 2.4. Combined Contamination of Mycotoxins

**Figure 2:**
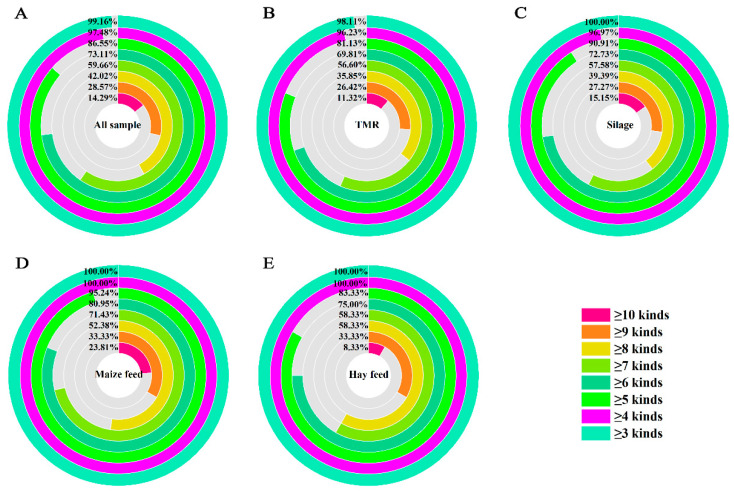
Combined contamination of mycotoxins in different feeds. (**A**) All samples; (**B**) TMR; (**C**) silage; (**D**) maize feed; (**E**) hay feed. Each colored circle represents the magnitude of the probability of coexistence of multiple mycotoxins in that type of feed.

Out of the 149 samples analyzed, 95.30% were found to be contaminated with three or more mycotoxins, which is shown in Figure 2A. Among them, 80.54% had the co-occurrence of 5 or more toxins, and 49.66% were co-exposed to 7 or more mycotoxins, and 7.38% were co-contaminated with 10 or more mycotoxins. As shown in Figure 2B–E, there were at least two mycotoxins found simultaneously in TMR maize feed. In the silage and hay feed, at least three mycotoxins were observed simultaneously. Additionally, there were more than 10 mycotoxins found simultaneously in TMR (3.77%), silage (6.06%), maize feed (9.38%), and hay feed (12.9%). The co-contamination of mycotoxins ranged from four to eight toxins simultaneously in the four feeds, seen in 79.26% of TMR, 81.82% of silage, maize feed at 71.86% and hay feed at 77.42%. Of the four feed samples, most of the feeds contained more than six mycotoxins (≥50.94%). Furthermore, the number of mycotoxins in maize feed and hay feed was higher than that in TMR and silage.

### 2.5. Correlation Analysis of Mycotoxins

**Figure 3:**
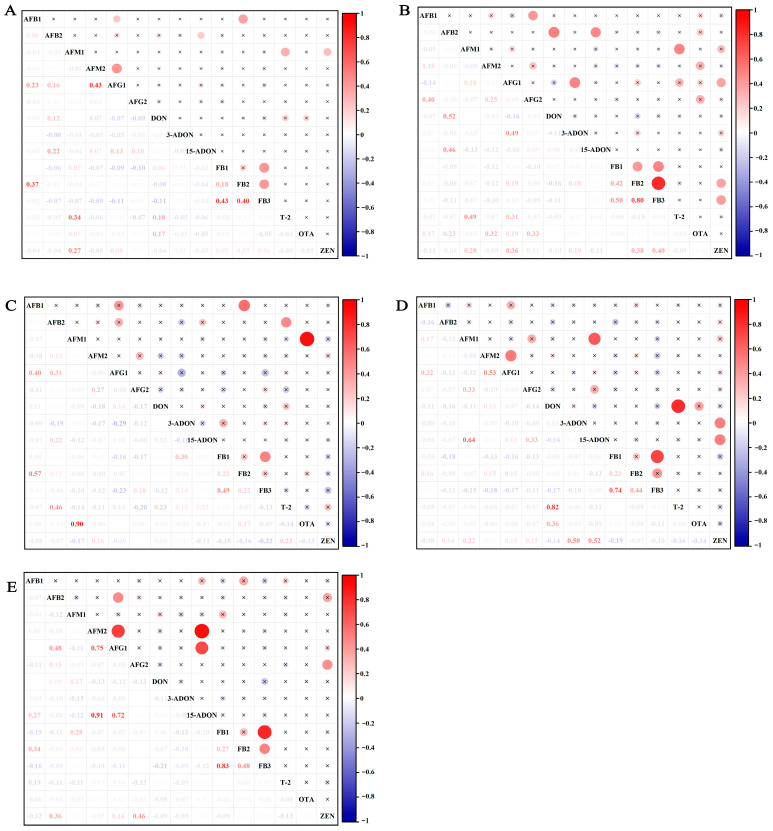
Heatmap of the correlation analyses of 15 mycotoxins in 149 feed samples. All samples (**A**); TMR (**B**); silage (**C**); maize feed (**D**); hay feed (**E**). Both the circle size and number indicate the strength of the correlation, while the blue-to-red gradient represents an increase in correlation from −1 to 1. An ‘×’ on the circle indicates no significance (*p* > 0.05), while the absence of an ‘×’ indicates significance (*p* < 0.05).

Correlation analysis of 15 mycotoxins across 149 feed samples revealed predominantly weak correlations and no significant correlation (R < 0.3, *p* ≥ 0.01) (Figure 3). However, moderate correlations (0.3 < R < 0.5, *p* < 0.01) were observed between FB3 and FB1/FB2, AFG1 and AFM2, AFM1 and T-2, and AFB1 and FB2 (Figure 3A). In TMR (Figure 3B), correlations with a moderate degree were detected between FB1 and FB2/FB3, ZEN and AFG1/FB2/FB3, T-2 and AFM1, AFG1 and 3-ADON, AFB2 and 15-ADON, and AFB1 and AFG2 (0.3 < R < 0.5, *p* < 0.01), while strong correlations were identified between DON and AFB2, and between FB2 and FB3 (R > 0.5, *p* < 0.01). In silage (Figure 3C), moderate correlations occurred between FB1 and FB3, and between T-2 and AFB2 (0.3 < R < 0.5, *p* < 0.01), whereas strong correlations were detected between AFM1 and OTA, and between FB2 and AFB1 (R > 0.5, *p* < 0.01). In maize feed (Figure 3D), a moderate correlation was observed between ZEN and 3-ADON (0.3 < R < 0.5, *p* < 0.01), while strong correlations emerged between FB1 and FB3, DON and T-2, 15-ADON and ZEN, and AFM2 and AFG1 (R > 0.5, *p* < 0.01). In hay feed (Figure 3E), moderate correlations were found between FB2 and FB3, and between AFB2 and AFG1 (0.3 < R < 0.5, *p* < 0.01), with strong correlations observed between AFM2 and AFG1/15-ADON, AFB1 and 15-ADON, and FB1 and FB3 (R > 0.5, *p* < 0.01). These findings highlight notable variations in mycotoxin correlations across different feed types, emphasizing the complexity of co-contamination patterns.

### 2.6. Interaction Analysis of Mycotoxins

A total of 10,430 mycotoxins were identified in 149 feed samples. Among the two mycotoxin combinations, the highest interaction rate (65.63%) was detected for DON + FB1 in maize feed. In all samples, DON + ZEN and FB1 + ZEN had the highest interaction rates of 51.01% and 43.62%, respectively.

**Figure 4:**
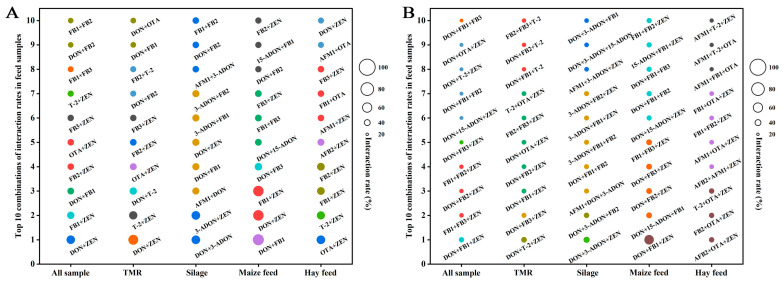
Interaction rates of 15 mycotoxins in all samples, TMR, silage, corn feed, and hay feed. (**A**,**B**) are combinations of 2 and 3 mycotoxins, showing all combinations with the top 10 interaction rates. Bubble size indicates the interaction rate of each mycotoxin combination.

In TMR, DON + ZEN (58.49%) and ZEN + T-2 (49.06%) presented the highest interaction rates. In silage, the highest interaction was for DON + 3-ADON and 3-ADON + ZEN at 51.52%, followed by DON + AFM1 (42.42%). In maize feed, in addition to DON+FB1, the interaction rate for DON + ZEN and FB1 + ZEN were the highest at 62.50%, followed by DON + FB3 (43.75%). The hay feed had the highest percentage of OTA + ZEN (51.61%), followed by T-2 + ZEN (48.39%) (Figure 4A). Across all analyzed samples, the DON + ZEN + FB1 combination exhibited the highest interaction rate at 31.54%, and then FB1 + FB2 + ZEN at 26.85%. In TMR, DON + ZEN + T-2 showed the highest interaction rate at 33.96%, and DON + ZEN+FB3 at 30.19%. In silage, DON + 3-ADON + ZEN showed the interactive rate (36.36%), with DON + 3-ADON + FB2 ranking second at 33.33%. In maize feed, DON + FB1 + ZEN stood out with a markedly high interaction rate of 56.22%, significantly exceeding all other combinations, which remained below 34.38%. In hay feed, three combinations, namely AFB2 + OTA + ZEN, FB2 + OTA + ZEN, and T-2 + OTA + ZEN, shared the highest interaction rate (32.26%), while all others were below 29.03%. These findings highlight significant variability in mycotoxin interactions across different feed matrices (Figure 4B).

In 149 feed samples, the interaction rate of combinations of two and three mycotoxins was higher, and the interactive rate of four or more mycotoxins was relatively lower. Table 4 shows that there were 1321 combinations of four mycotoxins, and DON + FB1 + FB2 + ZEN showed the highest interactive rate of 19.46%. The highest interaction rate among the five-mycotoxin combinations was 12.75% for the combination of 15-ADON + FB1 + FB2 + FB3 + ZEN, with a total of 2400 interaction combinations. A total of 2790 mycotoxin combinations were found that had six mycotoxins, but the interaction rate of all combinations was less than 8.72%. There was a total of 2022 mycotoxin combinations with seven mycotoxins, but the interaction rate of all combinations was less than 6.71%. A total of 1299 mycotoxin combinations were found for combinations of 8–11, but the interaction rate of all combinations was less than 4.03%. As the number of mycotoxins in the combination increased, the interaction rate gradually decreased. We found that the types of combinations in TMR and silage were similar according to the four feed interaction combinations, so we deduced that the mycotoxin contamination rate of TMR was mainly related to silage.

## 3. Discussion

In this study, we analyzed mycotoxin contamination of four typical dairy cow feeds (TMR, silage, maize, and hay feed), providing valuable insights into the challenges faced by livestock producers in managing feed safety. Mycotoxins are toxic secondary metabolites generated by specific fungi, which have the potential to contaminate feed materials and present substantial threats to both animal health and productivity. Especially in areas with humid climates, the problem of mycotoxin contamination is more prominent [19]. This study investigated mycotoxin contamination in feed and investigated the correlations and interactions among 15 mycotoxins. All the results found that four typical feeds, including TMR, silage, maize, and hay feed from Chinese pasture, were often found to be contaminated with mycotoxins, most frequently by ZEN, DON, OTA, FBs, T-2, and AFs. The average contamination level of all mycotoxins was well below the national safety limit, and the risk level was also low.

Aflatoxin contamination in feed is highest in warmer, more humid regions where mold growth is prevalent. Regulatory controls and enforcement vary widely, affecting the overall contamination status in each country [20]. In this research, 48 of 149 samples were positive for AFB1, accounting for 32.21% in all samples. Interestingly, the incidence of aflatoxin B1 was slightly above the range reported in the results of a 10-year survey of global feed mycotoxins, while the global prevalence of AFB1 was 23% compared with 17.1% in East Asia, which may be affected by the source of the sample and the climate [21]. In addition, 28.19% and 21.48% of samples were positive for AFB2, and AFG1 and AFG2, respectively. As dihydroxy derivatives of AFB2 and two other aflatoxins, G1 and G2 have not received extensive attention in aflatoxin detection and toxicology research due to their low toxicity [22]. In the regulation of feed hygiene standards promulgated by China, only a limit standard for AFB1 was designed. However, the maximum limit of aflatoxin in feed formulated by the USA and Canada is calculated by the sum of the contents of AFB1 + AFB2 + AFBG1 + AFG2. This study indicated that AFB1 + AFB2 + AFG1 + AFG2 co-occurrence should be paid some more attention in dairy cow feeds from pastures in regions of China. AFM1, as a hydroxylated metabolite of AFB1, is widely present in some milk of aflatoxin-contaminated cows [23,24,25], and was also detected about 34.90% of the cow feed in this experiment. In this study, AFB1 and AFM1 were the most heavily contaminated in silage, which ultimately led to a higher occurrence of AFB1 and AFM1 in the whole mixture. In silage, some fungal communities, along with their associated toxins, can survive and persist under harsh environmental conditions, such as low pH, elevated CO_2_ levels, and reduced O_2_ concentrations [26]. Moreover, positive correlations have been observed between silage temperatures exceeding ambient levels and the proliferation of fungi, leading to aerobic deterioration following silo opening [27], which ultimately leads to the production of mycotoxins.

Combined with the analysis of the detection rate and contamination level, the prevalence of *Fusarium* toxin in maize feed samples was relatively high, followed by silage and TMR; however, the contamination rate of *Fusarium* toxin in hay feed was relatively low. In TMR, silage, and maize feed, similar to our results, ZEN, DON and FBs appeared to be the most common mycotoxins and could be detected at higher concentrations [28]. Furthermore, the *Fusarium* toxins were detected in silage, and the detection rates of DON, ZEN, and FBs were 77%, 66%, and 80%, respectively [29]. Some findings reported that the incidence of DON was 78.13% in maize feed silage, with a mean level of 603 μg/kg and a maximum level of 711 μg/kg [30]. In our study, the detection rates of T-2 and DON were 46.31% and 66.44% in all the dairy feed samples. In Gintare’s study, 55.47% of the 119 silage samples contained T-2 and 52.11% contained DON, with DON being detected at a higher rate than T-2, which was consistent with the findings of this research [13]. However, our levels of T-2 detection rates were particularly low, with a mean T-2 level of 1.25 μg/kg in the mixed feed samples and an average T-2 concentration of less than1.04 μg/kg in the other three feeds. Additionally, 3-ADON and 15-ADON were also determined. In our experimental results, 35 samples were contaminated with 3-ADON and 31 samples were exposed to 15-ADON among the 67 feed samples [31]. An examination of 606 maize samples from China found that 99.83% of maize samples were contaminated by DON, and the incidence rate of 3-acetyl DON was 13.53% and that of 15-acetyl DON was 76.4% [32]. In this study, the level of 3-ADON and 15-ADON was higher than DON in silage, which may be attributed to the acetylation of DON during processing and storage of grain. Additionally, *Fusarium* could produce 3-ADON and 15-ADON, and the geographic location and climate in the region of the samples collected could also be considered [33]. On the other hand, the total fungal count increased during the silage fermentation process, with higher levels observed in post-fermentation samples compared with those taken prior to fermentation [34]. However, single samples’ exposure to multiple toxins was high and there was some species variation.

Food and animal feed are often contaminated by various mycotoxins or other fungal metabolites, and our research results verified this point. A few studies indicated that more than 51% of maize feed is contaminated by multiple mycotoxins in farms from Brazil [35]. Another study found that 97.6% of the feed samples in China, including silage, total mixed rations, and other feeds, were simultaneously detected to have multiple mycotoxins [36]. Our previous studies found that the co-exposure of multi-mycotoxins was detected in different grains, including rice, maize, soybean, and wheat flour [37]. In the 149 feed samples, almost all samples were co-contaminated by more than three mycotoxins. Strong correlations were found in TMR, between DON and AFB2, and between FB2 and FB3; in silage between AFM1 and OTA, and between FB2 and AFB1; in maize feed between FB1 and FB3, between DON and T-2, between 15-ADON and ZEN, and between AFM2 and AFG1; and in hay feed between AFM2 and AFG1 or 15-ADON, between AFB1 and 15-ADON, and between FB1 and FB3. Nevertheless, the correlations between the other mycotoxins were low, showing moderate or weak correlation. On the other hand, among the four typical feed samples, the combinations DON + ZEN, DON + FB1, FB1 + ZEN, OTA + ZEN, DON + 3-ADON, 3-ADON + ZEN, T-2 + ZEN, FB2 + ZEN, and DON + FB3 had higher interaction rates than the other combinations (≥43.75%). Our results are in line with other authors. Twarużek et al. [38] investigated the mycotoxin occurrence in Polish feed and found that the interaction rate of DON + ZEN was high (≥75%) and the correlation was very strong. A different study found DON + ZEN with a detection rate of 45% in maize silage samples [39]. Another study indicated that DON + ZEN (84%) was the most detected in maize samples [40]. A two-year survey showed the mycotoxin co-contamination of maize feed in Michigan, and the correlations between DON and its derivatives were relatively strong [41]. Furthermore, a very strong positive correlation was observed between AFB1 and AFB2, and the occurrence of FB1 and FB2 in feed samples from Asia-Oceania, and the correlation of a single mycotoxin largely depended on the commodity itself [42]. Another six kinds of Fusarium toxins were explored in wheat grains in China [43]. However, there has been little research on the correlation of mycotoxins between feed and raw materials. Comprehensive analyses of contamination levels, mixed contamination rates, correlations, and interactions showed that DON + ZEN, DON + FB1, FB1 + FB2, ZEN + FB1, AFM1 + OTA, and FB1 + FB2 were more closely related in the four typical feed samples.

Mycotoxins in feed easily accumulate in the serum of dairy cows and cause damage to their bodies, such as DON and ZEN [44]. Numerous studies have shown that compared with single mycotoxin exposure, the co-exposure of multi-mycotoxins might cause additional hazards [45]. It was found that AFB1 + DON or ZEN showed synergistic effects on PK-15 cells, and the toxicity of combined exposure was higher than that of single toxin exposure [46]. However, there are no regulations of food or feed related to the coexistence of mycotoxins in each country or organization. This study systematically analyzed the status of mycotoxin exposure in pasture feed of China, hoping to improve people’s awareness of mycotoxin safety prevention.

## 4. Conclusions

In this study, we analyzed mycotoxin contamination in 149 samples from four different types of feed collected from Chinese dairy farms, exploring the correlations and interactions among 15 mycotoxins. The results showed that mycotoxin contamination was prevalent across all four typical dairy feeds, with ZEN, DON, OTA, FBs, T-2, and AFs being the most common contaminants. Notably, the probability of a sample being contaminated with three or more mycotoxins was high, indicating significant co-contamination. Additionally, we observed correlations among DON, AFs, and ZEN, with the highest interaction rates (≥43.75%) found in combinations such as DON + ZEN, DON + FB1, FB1 + ZEN, OTA + ZEN, DON + 3-ADON, 3-ADON + ZEN, T-2 + ZEN, FB2 + ZEN, and DON + FB3 compared with other combinations. This study provides a clear understanding of the mycotoxin co-contamination status in dairy cattle feed in China and analyzes the correlations and potential cross-contamination among these mycotoxins. However, we focused on only 15 mycotoxins, and future research could further investigate additional mycotoxins and the underlying causes of their interactions.

## 5. Materials and Methods

### 5.1. Sampling

A total of 149 samples consisting of TMR (a mixture of silage, maize and hay feed in different proportions, *n* = 53), silage (feed produced by fermenting plant matter under anaerobic conditions until it is acidified, *n* = 33), maize feed (maize stalks after harvesting, *n* = 32), and hay feed (dried barley straw, oat straw, and cereal straw, *n* = 31) were collected from over 15 farms from 7 provinces in China during September 2022. Overall, the samples collected included 15 dairy farming enterprises from seven provinces including Heilongjiang, Jilin, Hebei, Shandong, Qinghai, Ningxia Hui, and Nei Mongol. Each sample weighed 500 g and was shipped to the laboratory in a sealed bag under temperatures between 0 and 4 °C. Once received, a randomly selected portion was processed for testing, while the remaining portion was stored at −20 °C.

### 5.2. Chemicals and Reagents

All organic solutions, including methanol (MeOH), acetonitrile (ACN), and ammonium acetate (CH_3_COONH_4_) for sample extraction and UPLC-MS/MS analysis were high-purity solvents (HPLC grade) purchased from Merck, Darmstadt, Germany. Fifteen mycotoxins (AFB1, AFB2, AFG1, AFG2, AFM1, AFM2, DON, 3-ADON, 15-ADON, FB1, FB2, FB3, T-2, OTA, and ZEN) as standard powders were acquired from Romer Labs, Newark, DE, USA. UPLC-MS/MS equipment was purchased from Waters, Milford, MA, USA. All remaining reagents were acquired from Sigma-Aldrich Pte. Ltd. (Shanghai, China).

### 5.3. Sample Extraction

Briefly, 2.00 g of the crushed feed sample was weighed accurately and transferred to a 50 mL centrifuge tube. A 10 mL aqueous water–acetonitrile (84:16, *v*/*v*) solution was supplied, vortexed for 30 min (70 rpm/min), sonicated for 30 min, and centrifuged at 5000 rpm for 5 min. Then the extracts were purified with a CLOVER SepMyco 100 Mass Spectrometry Multi-Toxin Clean-up Column (Beijing Clovertech Limited Company, Beijing, China), and 200 μL supernatant was put into a 2 mL centrifuge tube, with an 84:16 acetonitrile aqueous solution diluted to 1 mL. Before the UPLC-MS/MS analysis, the extracts were filtered through a 0.22 μm membrane. We chose to express mycotoxin concentrations (μg/kg) on a wet basis to better reflect the actual conditions of the feed as it is consumed by dairy cows.

### 5.4. UPLC-MS/MS Conditions

Analysis was performed on a C18 column (100 mm × 3.0 mm, 2.5 μm particle size) (Waters, Milford, MA, USA). The column temperature was kept at 40 °C. The injection volume was 3 μL and the flow rate was 0.4 mL/min. Eluent A was CH_3_COONH_4_ in H_2_O (5 mmol/L) for the remaining mycotoxins, and H_2_O with 0.1% HCOOH for the detection of three FBs, and Eluent B was CAN, using the following gradient elution program: The proportion of Eluent B was maintained at 10% from 0 to 3 min, then linearly increased from 70% to 90% between 3 and 5 min. It remained at 90% from 5 to 6 min, then linearly decreased from 90% to 10% between 6 and 6.1 min, and was held at 10% from 6.1 to 8 min. The electron spray ionization (ESI) detection conditions were as follows: atomization temperature, 500 °C; source temperature, 150 °C; atomization gas and auxiliary gas, 99.99% high-purity nitrogen; spray ion voltage (ESI+), 2500 V; spray ion voltage (ESI−), 1500 V; gas flow rate, 1000 L/h. Quantification of the target compounds was conducted using the multiple reaction monitoring (MRM) mode. Additional mass spectral parameters are provided in Table 5.

### 5.5. Statistical Analysis

The correlation cluster analysis and contamination levels in the feeds were analyzed using Origin 2021 (OriginLab, Northampton, MA, USA). The interactive analysis of mycotoxin co-contamination was conducted using a self-designed program written with Java, (V 1.0) for which a software copyright has been applied [37]. If the level of contaminants was below the limit of detection (LOD), it was recorded as “not detected”. For the purpose of calculating the mean value and correlation of the different toxins, non-detected data were substituted with half the LOD value (1/2 LOD). Additionally, in the mycotoxin interaction analysis, the interactions of individual mycotoxins were calculated primarily for positive samples (>LOD).

## Figures and Tables

**Table 1:** The linear ranges, R2, LOQ, and LOD for the targeted mycotoxins.

Mycotoxins	Linearity Range (μg/kg)	R^2^	LOQ (μg/kg)	LOD (μg/kg)
AFB1	0.1–100	0.9985	0.1	0.06
AFB2	0.1–100	0.9965	0.1	0.05
AFG1	0.1–100	0.9924	0.1	0.04
AFG2	0.1–100	0.9949	0.1	0.05
AFM1	0.1–200	0.9976	0.1	0.07
AFM2	0.1–200	0.9992	0.1	0.05
DON	10–200	0.9988	10	5.3
3-ADON	5–200	0.9969	5	2.4
15-ADON	5–200	0.9921	5	2.5
FB1	1–200	0.9952	1	0.48
FB2	5–200	0.9934	5	2.6
FB3	5–200	0.9926	5	2.1
T-2	0.1–200	0.9969	0.1	0.05
OTA	0.2–200	0.9988	0.2	0.16
ZEN	0.5–200	0.9979	0.5	0.2

**Table 2:** Average recovery of 15 mycotoxins from four typical feed matrices.

Mycotoxins	TMR (%)	Silage (%)	Maize Feed (%)	Hay Feed (%)
AFB1	70.7 ± 2.6	82.1 ± 2.9	98.3 ± 7.1	110.2 ± 9.8
AFB2	96.9 ± 7.8	110.2 ± 2.3	112.5 ± 8.6	90 ± 7.3
AFG1	77.3 ± 9.3	113.6 ± 4.5	117.4 ± 9.1	149.1 ± 5.4
AFG2	88.6 ± 6.8	86.9 ± 8.4	89.6 ± 6.3	79.7 ± 6.1
AFM1	149.8 ± 4.1	78.3 ± 3.1	99.4 ± 4.1	89.9 ± 2.8
AFM2	106.3 ± 6.7	113.4 ± 2.6	87.5 ± 4.2	73.8 ± 2.6
DON	80.2 ± 5.1	86.3 ± 1.9	149.4 ± 8.9	118.1 ± 5.8
3-ADON	98.7 ± 1.9	108.7 ± 4.5	110.8 ± 7.7	108.4 ± 4.7
15-ADON	110.2 ± 7.6	1.8.2 ± 5.9	79.7 ± 5.6	116.3 ± 6.7
FB1	117.8 ± 2.5	118.4 ± 5.4	82.9 ± 4.2	107.5 ± 5.9
FB2	80.9 ± 5.4	110.1 ± 7.1	88.8 ± 6.7	80.9 ± 4.8
FB3	90.8 ± 3.7	89.5 ± 4.8	112.6 ± 2.1	90.4 ± 6.1
T-2	110.5 ± 4.1	97.6 ± 0.9	89.5 ± 3.5	118.8 ± 6.2
OTA	107.1 ± 9.4	99.7 ± 4.8	88.3 ± 2.7	103.5 ± 7.2
ZEN	99.4 ± 4.6	94.6 ± 6.2	91.6 ± 9.8	82.9 ± 9.1

Here, 2.00 g of crushed feed samples was weighed and added to 10 mL an aqueous water–acetonitrile (84:16, *v*/*v*) solution and standardized working solutions of 15 mycotoxins (three different concentration levels, namely low, medium, and high, were added), and their respective mean recoveries were calculated on the basis of the percentage of the measured values versus the theoretical values. The concentrations of mycotoxins were 4 ng/mL, 40 ng/mL, and 80 ng/mL.

**Table 3:** Contamination levels of the 15 tested mycotoxins in TMR, silage, maize, and hay feed.

Mycotoxins (μg/kg)	TMR (*n* = 53)		Silage (*n* = 33)		Maize Feed (*n* = 32)		Hay Feed (*n* = 31)	
	Average	Range	Average	Range	Average	Range	Average	Range
AFB1	0.20	0.12~1.33	3.53	0.16~53.69	0.10	0.13~0.72	0.38	0.13~4.35
AFB2	0.64	0.67~12.29	2.16	0.32~26.32	0.81	0.06~8.58	0.46	0.08~6.21
AFG1	0.92	0.38~14.32	0.44	0.48~8.62	0.25	0.44~3.28	0.41	0.14~2.71
AFG2	0.13	0.49~2.12	0.39	0.13~3.83	1.14	0.08~14.55	0.27	0.14~5.42
AFM1	2.97	0.53~36.22	9.52	0.14~57.37	8.88	0.13~93.99	3.32	0.19~47.76
AFM2	0.25	0.28~6.49	1.74	0.16~18.52	1.38	0.13~16.27	0.36	0.12~3.79
DON	32.63	0.16~328.25	10.14	0.26~75.35	36.75	0.14~348.69	10.82	6.36~58.79
3-ADON	14.05	0.69~285.90	77.65	0.14~725.78	23.12	0.36~476.24	6.81	0.76~94.82
15-ADON	11.41	2.42~149.94	13.68	0.13~142.18	16.30	2.11~137.88	4.39	2.94~54.94
FB1	142.81	1.18~1347.28	62.40	1.15~743.30	58.28	2.37~430.79	70.24	0.24~584.06
FB2	7.16	0.35~58.52	13.21	0.25~252.47	7.03	0.13~69.87	21.38	0.34~186.69
FB3	5.04	0.42~45.89	8.08	0.13~53.92	12.20	1.38~63.87	12.14	0.42~100.83
T-2	1.25	0.12~27.74	0.20	0.12~1.37	0.66	0.22~16.19	1.04	0.13~11.88
OTA	0.59	0.14~6.19	1.63	0.14~29.48	3.03	0.17~85.43	2.53	0.38~53.22
ZEN	70.61	0.63~696.18	14.86	2.32~61.14	11.82	0.08~51.67	13.94	0.27~198.33

**Table 4:** The top three combinations in terms of the interaction rate in 149 feed samples, included those with interactions of 4, 5, 6, 7, and 8–11 contaminants.

Amount of Toxins	Number of Combinations	Toxin Combination (Top 3 Combinations in Terms of Interaction Rate)	Frequency	Interaction Rate (%)
4 kinds	1321	DON + FB1 + FB2 + ZEN	29	19.46
		DON + FB1 + FB3 + ZEN	27	18.12
		FB1 + FB2 + FB3 + ZEN	26	17.45
5 kinds	2440	DON + FB1 + FB2 + FB3 + ZEN	19	12.75
		DON + 15-ADON + FB1 + FB2 + ZEN	17	11.41
		DON + 15-ADON+ FB2 + T-2 + ZEN	16	10.74
6 kinds	2790	DON + 15-ADON + FB2 + FB3 + T-2 + ZEN	13	8.72
		DON + FB1 + FB2 + FB3 + T-2 + ZEN	12	8.05
		DON + 15-ADON + FB1 + FB2 + FB3 + T-2	11	7.38
7 kinds	2022	DON + 15-ADON + FB1 + FB2 + FB3 + T-2 + ZEN	10	6.71
		DON + 15-ADON + FB2 + FB3 + T-2 + OTA + ZEN	8	5.37
		DON + FB1 + FB2 + FB3 + T-2+ OTA + ZEN	8	5.37
8–11 kinds	1299	DON + 3-ADON + 15-ADON + FB1 + FB2 + FB3 + T-2 + ZEN	6	4.03
		DON + 15-ADON + FB1 + FB2 + FB3 + T-2 + OTA + ZEN	6	4.03
		DON + 3-ADON + 15-ADON + FB1 + FB2 + FB3 + T-2 + OTA	4	2.68

**Table 5:** Mass spectrometry parameters for the detection of the tested 15 mycotoxins.

Mycotoxins	Retention Period (min)	Parent Ion (*m*/*z*)	Daughter Ion (*m*/*z*)	Impulse Voltage (ev)	Ion Source
AFB1	3.32	313.24	241.16 */284.97	36/22	ES+
AFB2	3.17	315.22	287.14 */259.12	28/26	ES+
AFG1	2.83	329.22	273.12 */229.17	38/22	ES+
AFG2	3.04	331.23	284.99 */241.10	42/24	ES+
AFM1	3.18	329.22	243.08 */199.86	42/26	ES+
AFM2	3.03	331.22	189.05 */245.09	40/30	ES+
DON	1.99	297.1	249.1 */231.1	13/10	ES+
3-ADON	2.71	339.23	231.16 */213.15	16/12	ES+
15-ADON	2.66	356.03	339.02 */320.94	12/6	ES+
FB1	2.97	722.39	334.38 */352.38	38/34	ES+
FB2	3.41	706.39	336.39 */354.39	36/32	ES+
FB3	3.23	706.39	336.39 */354.39	36/32	ES+
T-2	4.78	484.35	305.18 */185.13	14/18	ES+
OTA	2.90	404.29	239.03 */221.03	34/22	ES+
ZEN	4.19	317.24	175.05 */131.03	26/24	ES−

Note: * are quantitative ions.

## Data Availability

The original contributions presented in this study are included in the article/Supplementary Materials. Further inquiries can be directed to the corresponding author(s).

## Supplementary Materials

The following supporting information can be downloaded at: https://www.mdpi.com/article/10.3390/toxins17030137/s1, Table S1: Exceedance rates of 15 target mycotoxins in TMR, silage, maize and hay feed samples.
