# Supplementary material for: Occurrence of Co-Contamination and Interaction of Multi-Mycotoxins in Dairy Cow Feed in China
Source: Toxins (Basel). 2025 Mar 14;17(3):137. doi: 10.3390/toxins17030137 (PMC11945585; doi:10.3390/toxins17030137)
Supplement: Supplementary file 1 [file toxins-17-00137-s001.zip › toxins-3512279-supplementary.pdf]

Supplementary Materials: Occurrence of Co-Contamination and Interaction of Multi-Mycotoxins in Dairy Cow Feed in China

Table S1. Exceedance rates of 15 target mycotoxins in TMR, silage, maize and hay feed samples.

Mycotox-ins	MRLs (μg/kg)	TMR (n=53)		Silage (n=33)		Maize feed (n=32)		Hay feed (n=31)	
		Amount (n)	Rate (%)	Amount (n)	Rate (%)	Amount (n)	Rate (%)	Amount (n)	Rate (%)
AFB1	30	-	-	2	6.06	-	-	-	-
OTA	100	-	-	-	-	-	-	-	-
ZEN	1000	-	-	-	-	-	-	-	-
DON	5000	-	-	-	-	-	-	-	-
T-2	500	-	-	-	-	-	-	-	-
FB1+FB2	60000	-	-	-	-	-	-	-	-

“-” indicates that mycotoxin-contaminated samples did not exceed the MRLs.
